# Genetic Control of Differential Acetylation in Diabetic Rats

**DOI:** 10.1371/journal.pone.0094555

**Published:** 2014-04-17

**Authors:** Pamela J. Kaisaki, Georg W. Otto, Joanna F. McGouran, Amine Toubal, Karène Argoud, Helen Waller-Evans, Clare Finlay, Sophie Caldérari, Marie-Thérèse Bihoreau, Benedikt M. Kessler, Dominique Gauguier, Richard Mott

**Affiliations:** 1 The Wellcome Trust Centre for Human Genetics, University of Oxford, Headington, Oxford, United Kingdom; 2 Target Discovery Institute, Nuffield Department of Medicine, University of Oxford, Oxford, United Kingdom; 3 INSERM, U872, Cordeliers Research Centre, Paris, France; 4 Institute of Cardiometabolism & Nutrition, ICAN, Pitié-Salpêtrière Hospital, University Pierre & Marie-Curie, Paris, France; 5 National Genotyping Centre, Evry, France; Institute of Enzymology of the Hungarian Academy of Science, Hungary

## Abstract

Post-translational protein modifications such as acetylation have significant regulatory roles in metabolic processes, but their relationship to both variation in gene expression and DNA sequence is unclear. We address this question in the Goto-Kakizaki (GK) rat inbred strain, a model of polygenic type 2 diabetes. Expression of the NAD-dependent deacetylase Sirtuin-3 is down-regulated in GK rats compared to normoglycemic Brown Norway (BN) rats. We show first that a promoter SNP causes down-regulation of Sirtuin-3 expression in GK rats. We then use mass-spectrometry to identify proteome-wide differential lysine acetylation of putative Sirtuin-3 protein targets in livers of GK and BN rats. These include many proteins in pathways connected to diabetes and metabolic syndrome. We finally sequence GK and BN liver transcriptomes and find that mRNA expression of these targets does not differ significantly between GK and BN rats, in contrast to other components of the same pathways. We conclude that physiological differences between GK and BN rats are mediated by a combination of differential protein acetylation and gene transcription and that genetic variation can modulate acetylation independently of expression.

## Introduction

Post-translational modification (PTM) of proteins is one means by which an organism can alter protein function independently of transcription and translation. There are over 400 characterised PTMs, including phosphorylation, acetylation, methylation, ubiquitination and SUMOylation. In this study, we focus on protein acetylation, which is regulated by complex mechanisms involving families of acetyltransferases and deacetylases. Acetylation of enzymes may affect their activity, and is a mechanism used by cells to make metabolic adaptations, possibly through the coordination of pathways such as glycolysis, gluconeogenesis, citrate cycle, fatty acid metabolism, urea cycle, and glycogen metabolism [1]. Many of the essential enzymes required for metabolite processing in liver are acetylated [2]. As a consequence, variation in protein acetylation is expected to affect diseases such as diabetes and the metabolic syndrome (Reaven's syndrome).

Animal models of diabetes can reveal causal relationships between altered protein acetylation and impaired glucose homeostasis. The Goto-Kakizaki (GK) rat is an established model of spontaneous non-obese Type 2 diabetes, which has elevated blood glucose and peripheral insulin resistance similar to human Type 2 diabetes [3]. The genetic basis of metabolic and hormonal anomalies in the GK rat has been mapped in crosses between GK and non-diabetic Brown Norway (BN) rats which identified quantitative trait loci (QTL) linked to diabetes-related phenotypes such as glucose intolerance [4]. The GK rat is also a model for the metabolic syndrome, because it exhibits traits of salt-induced hypertension, increased adiposity, glucose intolerance, and hyperlipidemia [5]–[8].

Previous genome-wide liver transcriptome analyses in rat strains [9] identified significantly down-regulated expression of the NAD-dependent deacetylase sirtuin 3 (*Sirt3*) in GK compared to BN rats. Sirtuins influence insulin sensitivity and energy homeostasis and possibly ageing. There is controversy over their role in ageing [10], but it is known that they mediate metabolic adaptation to stress and changes in nutrition [11].

The transcriptional difference between GK and BN was confirmed in a further microarray study [6] of a congenic rat strain carrying a large section (170 Mb) of GK chromosome 1 associated with glucose intolerance, where *Sirt3* is localised, on a BN genetic background (1consomic). GK alleles in this region are associated with lower *Sirt3* expression (p = 0.0030). *Sirt3* is likely to affect metabolism and may account for the observed effect because it is a key regulator of mitochondrial protein acetylation levels [12].

Due to the potential importance of differential expression of *Sirt3* between BN and GK rats, we undertook a series of experiments to first validate and then fine-map the difference in *Sirt3* transcript levels to discover the cause of this variation, and finally to explore the effects of lower *Sirt3* deacetylase activity on protein acetylation in liver, and determine whether differences in lysine acetylation of metabolic enzymes sheds light on the pathophysiology of the GK rat. We identify alterations in liver protein acetylation and gene transcription that may contribute to physiological differences between GK and BN rats.

## Results

### Transcription regulation of *Sirt3* in congenic strains of the GK rat

**Figure 1:**
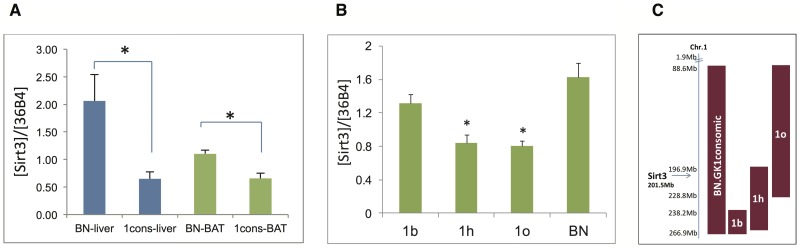
Validation of *Sirt3* transcript levels. QRT-PCR of *Sirt3* levels (A) comparing BN control rat and congenic strain 1consomic (which has GK chromosome 1 on a BN genetic background), in liver (blue) and BAT (green); (B) left, comparing BAT from BN and three congenic strains, two carrying GK allele of *Sirt3* (1o and 1h), and one with BN allele of *Sirt3* (1b); (C) diagram of BN.GK chromosome 1 congenic strains used, with maroon bars representing region of GK chromosome 1 introgressed onto BN background. (Genomic location of *Sirt3* is indicated.) Results are corrected for expression of housekeeping gene 36B4.

We performed genome-wide gene expression analysis using Illumina expression microarrays, on white adipose tissue, kidney, skeletal muscle, liver and brown adipose tissue (BAT) from BN and 1consomic rats, and found 1.6-fold higher transcript levels of *Sirt3* in BAT from BN rats compared to 1consomic rats (p = 6.7×10^−5^), and similar results in liver, consistent with our earlier finding obtained with Affymetrix arrays in liver from the same congenic and control rats [9]. *Sirt3* is the tenth most significantly differentially expressed gene (DEG) out of 92 DEG in the 1consomic interval in BAT, and twelfth most differentially expressed gene in liver (out of 100 DEG). The difference in *Sirt3* expression appears to be specific to liver and BAT, because it was not present in white adipose, kidney or skeletal muscle. We performed qRT-PCR in order to validate these differences in liver and BAT from BN and 1consomic rats. Expression of *Sirt3* was significantly lower in both tissues from 1consomic compared to BN (p<0.05) (Figure 1A).

To fine-map this variation, we used qRT-PCR to compare *Sirt3* transcription in BAT from three BN.GK congenic strains of rat chromosome 1 containing either the GK allele of *Sirt3* (strains 1h and 1o) with BN or the BN allele of *Sirt3* (strain 1b). Figure 1B, right, illustrates these congenic strains, and shows that compared to BN and congenic 1b, *Sirt3* expression was lower (p<2.0×10^−4^) in strains 1h and 1o. These results confirm that GK alleles nearby *Sirt3* down-regulate its transcription in cis.

### Identification and functional analysis of *Sirt3* polymorphisms in the GK rat

To identify the *cis*-acting variants affecting *Sirt3* transcription, we compared the sequences of the *Sirt3* gene, including its promoter, in GK and BN in the interval 1:201019865-201045697. We detected 64 intronic SNPs, 3 intronic insertions, 3 intronic deletions, one known synonymous coding SNP (rs13449840), and one SNP in the 5′-UTR, which was within the *Sirt3* promoter (1:201044229_C/T). We investigated the consequences of segregating polymorphisms using the Variant Effect Predictor [13]) (Table S1) but no obviously functional coding variants were found.

**Figure 2:**
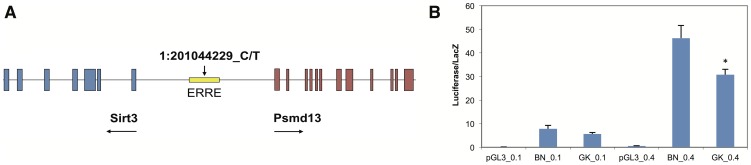
*Sirt3* promoter variant and luciferase assay. (A) Bidirectional promoter of *Sirt3* and *Psmd13*. One single nucleotide polymorphism (1:201044229_C/T) was identified when comparing sequence of GK promoter with BN. (B) Luciferase reporter assay results for transfection of hepatoma cell line. BN or GK allele of *Sirt3* promoter was cloned into pGL3-basic vector upstream of luciferase gene, and transfected into Hep3B cells at two levels, 0.1 and 0.4 ug/well. Results are corrected for transfection efficiency by co-transfection with beta-galactosidase vector, and represent two-three independent transfections, which were run in triplicate wells.

The 646 bp *Sirt3* promoter is bi-directional, and lies between the transcriptional start site (TSS) of *Sirt3* and the TSS of a neighboring gene encoding the proteasome non-ATPase regulatory subunit 13 (*Psmd13*) (Figure 2A) [14]. Expression of *Psmd13* was not altered in liver and BAT of GK and BN rat. The *Sirt3* promoter SNP occurs within a predicted binding site for the estrogen-related receptor response element (ERRE) transcription factor, which is known to activate *Sirt3* transcription in the mouse [15]. In order to test whether the variant affected function of the ERRE, we cloned both the BN and GK alleles of the promoter sequence into luciferase reporter construct pGL3-basic. No difference in luciferase activity was observed when HEK293T kidney cells were transfected at two concentrations with the GK promoter compared to BN control (Figure S1). However, when transfected into Hep3B hepatocyte cells, luciferase activity driven by the GK promoter at the higher level of transfection was significantly less than that of BN (p<0.05) (Figure 2B). We conclude that the GK promoter variant in *Sirt3* is the likely cause of the *cis*-regulated transcriptional downregulation of the GK allele, and that this effect is tissue-specific.

### RNA-sequencing

We sequenced mRNA from the liver and BAT of GK and BN rats, and identified differentially expressed transcripts in each tissue (Tables S2 and S3). RNA-sequencing re-confirmed that *Sirt3* is down-regulated in both BAT (GK 47% lower than BN) and liver (37% lower) from GK rats compared to BN. KEGG pathway enrichment analysis was then performed (Tables S4 and S5). In BAT, the most significantly over-represented pathways that differentiated GK and BN rats were “Metabolic pathways” (p = 3.08×10^−13^) and “Oxidative phosphorylation” (p = 1.63×10^−9^). Digestion and absorption of protein and fat are also over-represented in BAT, as well as fatty acid metabolism and PPAR signaling pathway. In liver, KEGG analysis again highlights “Metabolic pathways” as the most significantly over-represented pathway amongst the differentially expressed genes (p = 8.41×10^−14^). Cytochrome P450 metabolism, PPAR signaling and steroid hormone biosynthesis are among the top five significantly over-represented pathways in liver. Because *Sirt3* is a target of PPARG-coactivator1-alpha, and therefore involved in PPAR-gamma signaling, it is not surprising that PPAR signaling is affected [15].

### Proteome-wide analysis of liver protein acetylation

Since *Sirt3* is the major deacetylase enzyme in mitochondria, we reasoned that lower levels of *Sirt3* in GK liver would increase lysine-acetylation of proteins. Therefore, we determined global liver protein acetylation in GK and BN rats. First, in order to enrich for acetylated proteins, an equal amount of liver protein extract from GK and BN rats was digested with trypsin, then subjected to immunoprecipitation using pan-acetyllysine antibodies. These enriched peptides were analysed by liquid chromatography tandem mass spectrometry (LC-MS/MS). The 89 differentially acetylated peptides we identified with >95% confidence are listed in Table S6, and examples are shown in Figure S2. Unfortunately, we could not determine global lysine acetylation in BAT, possibly due to insufficient tissue.

**Figure 3:**
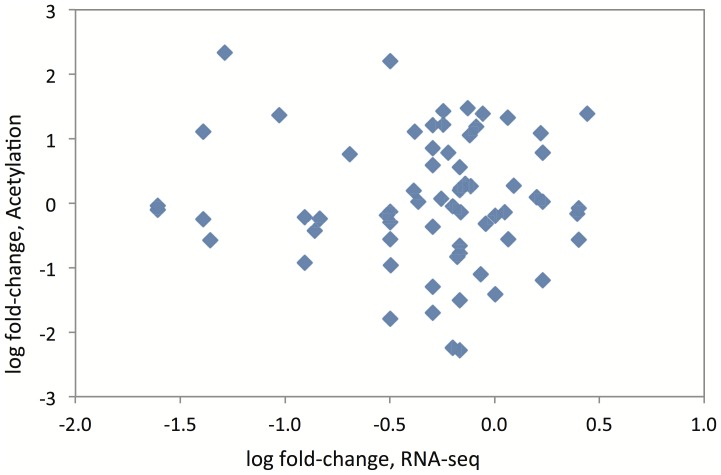
Scatter-plot of log fold-change of lysine acetylation and fold-change of RNA-sequencing. Each point represents one protein/transcript. y-axis: log2 of fold change in protein acetylation between GK and BN rats. x-axis: log2 of fold-change in mRNA sequence counts between GK and BN rats.

As the measurement of acetylated peptides does not distinguish between differing levels of protein and differing levels of acetylation, we used our transcriptomic data to test if the acetylation differences were correlated with variable gene expression, which we measured on the level of gene transcription. Absence of differential gene expression would suggest that observed acetylation differences are due to differential protein acetylation. Transcript measurements for each lysine-acetylated protein are given in Table S6. Transcripts for 28% of the acetylated proteins are differentially expressed at FDR p<0.05, but for 91% of the peptides, there is either no change in transcript level, or it is in the opposite direction to the change in acetylation. When fold-change of acetylation is plotted against fold-change of RNA-seq, no correlation is observed (Figure 3). Therefore, most of the differences measured by mass-spectrometry are likely to be due to varying acetylation levels rather than amount of protein. Eighteen genes encoding known acetylated proteins have significantly different gene expression between BN and GK or 1consomic liver (see Table 1), but none were significantly differentially acetylated (malate dehydrogenase 1, but not malate dehydrogenase 2, is differentially acetylated). We do not know whether the proteins in Table 1 are not acetylated in GK or BN rat liver, or whether they are acetylated but below our level of detection.

**Table 1:** Illumina liver transcripts significantly differentially expressed between GK and BN or 1Consomic strain rats.

Gene Symbol	Description	GK/BN log2 fold-change	GK/BN FDR	1Cons/BN log2 fold-change	1Cons/BN FDR
*Phf20*	PHD finger protein 20	0.68	**0.0000**	−0.21	0.146
*Asl*	argininosuccinate lyase	−1.40	**0.0000**	0.15	0.666
*Mdh2*	malate dehydrogenase 2, NAD (mitochondrial)	0.41	**0.0002**	−0.30	**0.027**
*Spi1*	spleen focus forming virus (SFFV) proviral integration oncogene spi1	0.84	**0.0003**	0.08	0.819
*Taf5l*	TAF5-like RNA polymerase II, p300/CBP-associated factor (PCAF)-associated factor	−0.59	**0.0005**	0.63	**0.008**
*Ncoa1*	nuclear receptor coactivator 1	−0.42	**0.0022**	−0.10	0.609
*Dscc1*	defective in sister chromatid cohesion 1 homolog (S. cerevisiae)	0.39	**0.0044**	0.26	0.152
*Ctbp1*	C-terminal binding protein 1	0.28	**0.0047**	−0.20	0.145
*Pole4*	polymerase (DNA-directed), epsilon 4 (p12 subunit)	0.26	**0.0071**	0.09	0.509
*Yeats4*	YEATS domain containing 4	0.32	**0.0097**	−0.09	0.621
*Kat2a*	K(lysine) acetyltransferase 2A	0.42	**0.0138**	0.09	0.745
*Gata2*	GATA binding protein 2	−0.28	**0.0188**	0.00	0.988
*Por*	P450 (cytochrome) oxidoreductase	−0.55	**0.0347**	0.16	0.695
*Mbd3*	methyl-CpG binding domain protein 3	0.28	**0.0361**	0.29	0.119
*Bag6*	BCL2-associated athanogene 6	−0.23	**0.0363**	0.08	0.606
*RGD1563945*	similar to mKIAA0215 protein	−0.14	0.1694	−0.30	**0.037**
*Bmi1*	Bmi1 polycomb ring finger oncogene	0.18	0.5394	0.79	**0.039**
*Eid1*	EP300 interacting inhibitor of differentiation 1	0.02	0.9189	0.49	**0.040**

Gene ontology descriptions relating to protein acetylation or deacetylation are shown. Transcripts with False Discovery Rates (FDR)<0.05 are in bold.

Most of the differentially acetylated peptides between GK and BN liver (Table S6, Figure S2), are enzymes in key metabolic pathways, as listed below. Thirty-six of the 89 proteins listed were also found to be acetylated in a study of *Sirt3*-knockout mice, providing evidence that they are targets of *Sirt3* [16]. Note that proteins annotated “BN only” or “GK only” might still be acetylated below the level of detection for the experimental approach used.

#### (i) Glycolysis and gluconeogenesis

Peptides from glyceraldehyde 3-phosphate dehydrogenase (*Gapdh*) were acetylated between 2.3- and 2.7-fold more in GK than BN liver. One acetylated peptide of dihydrolipoamide dehydrogenase (*Dld*), a component of pyruvate dehydrogenase complex, is only detected in GK liver, and another *Dld* derived peptide is about 10% less acetylated in GK than BN. Acetylated phosphoglycerate kinase 2 was detected in BN only. Other acetylated proteins with the KEGG term glycolysis/gluconeogenesis include alcohol dehydrogenase (*Adh*), with one peptide 2-fold more acetylated in GK, and one slightly less in GK than BN, and lactate dehydrogenase, where an LdhC peptide is acetylated in GK only and an LdhA peptide acetylated in BN only.

#### (ii) TCA cycle

Acetylation of aconitate hydrolase (*Aco*) is 2-fold higher in GK liver than in BN. Dihydrolipoyllysine-residue succinyltransferase (*Dlst*) and one peptide of dihydrolipoamide dehydrogenase (*Dld*), which are components of the α-ketoglutarate dehydrogenase complex in TCA cycle, are only acetylated in GK liver. Succinyl-CoA synthetase alpha subunit (*Suclg1*) is 2.6-fold more acetylated in GK than BN livers. Both succinate dehydrogenase (*Sdh*), a known target of *Sirt3*, and fumarate hydratase, were only acetylated in GK. Malate dehydrogenase 1 (*Mdh1*) was only acetylated in BN liver (this is the cytosolic isoform involved in the malate-aspartate shuttle). Peptides from different subunits of ATP synthase in oxidative phosphorylation are also acetylated: ATP synthase subunit A is only acetylated in GK, ATP synthase subunit B is acetylated in both GK and BN, and ATP synthase subunit D is twice as acetylated in GK as BN.

#### (iii) Pentose phosphate pathway (hexose monophosphate shunt)

Components of this pathway, which is the major source of NADPH required for anabolic processes, were acetylated. For example, UDP-glucose 6-dehydrogenase was only acetylated in GK liver. Transketolase (*Tkt*) was 2.6-fold more acetylated in GK than BN liver.

#### (iv) Fatty acid metabolism

These pathways include acetyl-CoA acyltransferase (*Acaa2*), an enzyme in fatty acid beta-oxidation and elongation, which has 2 acetylated peptides that are only detected in GK liver. One peptide of mitochondrial acetyl-CoA acetyltransferase *Acat1* is more highly acetylated in GK rat liver than in BN, but other peptides from *Acat1* and a peptide from cytosolic *Acat2* are only acetylated in BN liver. Mitochondrial medium-chain acyl-CoA dehydrogenase (*Acadm*) and enoyl-CoA delta isomerase 1 (*Eci1*) are only acetylated in GK liver. Echs1 (enoyl-CoA hydratase/3-ketoacyl thiolase 1, mitochondrial) is acetylated about equally in GK and BN. Several peptides of fatty acid-binding protein, important in fat transport and PPAR signaling, are acetylated, and one of these is 2.7-fold more acetylated in BN than in GK. Diazepam binding inhibitor (*Dbi*), which plays a role in acyl-CoA metabolism and mitochondrial steroidogenesis, is 1.5-fold more acetylated in BN than GK.

#### (v) Amino acid metabolism

Lysine ketoglutarate reductase, in lysine degradation, is only acetylated in GK. Similarly, aspartate aminotransferase (*Got2*) is only acetylated in GK, as was choline dehydrogenase (*Chdh*) and dimethylglycine dehydrogenase (Dmgdh), which are involved in glycine, serine and threonine metabolism, and glutamic-oxaloacetic transaminase 2 (*Got2*). Formimidoyltransferase-cyclodeaminase (*Ftcd*), an enzyme of histidine and one carbon metabolism, is only acetylated in BN. Omega-amidase (*Nit2*), which is involved in alanine, aspartate and glutamate metabolism, is acetylated 2-fold more in BN than in GK liver. There are several enzymes involved in amino acid metabolism that are acetylated, but differ less than 25% in their acetylation between BN and GK. These include aldehyde dehydrogenase 9A1 (*Aldh9a1*), which participates in degradation of many amino acids; arginase 1, betaine-homocysteine S-methyltransferase (*Bhmt*), which functions in methionine, cysteine, glycine, serine and threonine metabolism, and S-adenosylhomocysteine hydrolase (*Ahcy*), which is involved in cysteine and methionine metabolism.

#### (vi) Purine and pyrimidine pathway

This includes adenosine kinase (*Adk*) and adenylate kinase (*Ak*), which are only acetylated in GK liver. Two peptides of carbamoyl-phosphate synthetase I (*Cps1*) are acetylated, one at about equal levels in BN and GK, and one that is twice as acetylated in BN as compared to GK. Carbamoyl-phosphate synthetase 2/aspartate transcarbamylase/dihydroorotase (*Cad*) is only acetylated in GK. Dihydropyrimidinase (*Dpy*) is very similar, with two acetylation sites detected, one about equally acetylated and one that is only acetylated in BN (Table S6).

Thus, as in other studies of protein acetylation, we find a wide range of metabolic pathways whose enzymes are affected by this modification. However, as lysine acetylation can either activate or inhibit an enzyme's activity, the direction of effect must be determined empirically for each protein. Therefore, we cannot predict the overall consequence of differential acetylation for the majority of these pathways.

**Figure 4:**
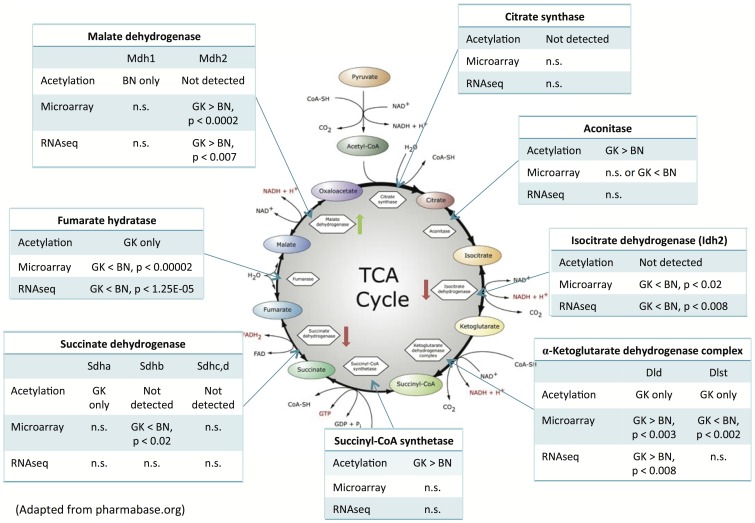
Lysine acetylation and mRNA expression in components of TCA cycle. Green arrow pointing up (next to malate dehydrogenase) indicates acetylation causes increase in enzyme activity. Red arrow pointing down (next to isocitrate dehydrogenase and succinate dehydrogenase) refers to decrease in enzyme activity with acetylation.

However, the citrate (TCA) cycle does contain enzymes for which the effect of acetylation has been directly determined, and by combining transcriptomic and proteomic data, we can predict the effects on this pathway due to acetylation rather than amount of protein. Additionally, we observe some TCA proteins that are not acetylated are nonetheless differentially expressed, which can also affect enzyme activity. Thus, the TCA pathway serves as a model for integrating the transcriptome and proteome, in order to give a more complete view of differences between the diabetic GK and control BN rat liver (Figure 4).

### Liver transcription regulation of acetyltransferases and deacetylases

We also examined transcription patterns of genes encoding acetyltransferases and deacetylases in our Illumina transcriptomic data that were identified using Gene Ontology terms. We did so in order to determine whether the differences that we measured in lysine acetylation are part of a general trend toward altered acetylation, or specific to the down-regulation of *Sirt3* (Tables 2 and 3). Nineteen acetyltransferases and eleven deacetylases were significantly differentially expressed between BN and GK, though the annotation may be incomplete. For instance, *Sirt3* was not originally annotated on the Illumina array as a deacetylase. Besides *Sirt3*, there are five other deacetylases that show significantly lower transcript level in GK than BN livers: MACRO domain containing 1 (*Macrod1*), amidohydrolase domain containing 2 (*Amdhd2*), and histone deacetylases *Hdac3*, *Hdac5*, and *Hdac11* (however, the substrates of *Macrod1* and *Amdhd2* are not proteins). Deacetylases that are overexpressed in GK (or 1consomic) when compared to BN are N-deacetylase/N-sulfotransferase (*Ndst1*), with target heparan sulfate (a linear polysaccharide), Phosphatidylinositol glycan anchor biosynthesis, class L (*Pigl*), with target N-acetyl-D-glucosaminylphosphatidylinositol (a glycerophospholipid), arylacetamide deacetylase (*Aadac*), and ataxin 3 (*Atxn3*). Acetyltransferases that are significantly different between BN and GK include enzymes that acetylate xenobiotics (*Nat1*, higher in GK, and Nat2, lower in GK), metabolites (*Acat3*, *Gnpnat1* and *Crat*, higher in GK; *Gnpat*, lower in GK), phospholipid (*Lpcat1*, lower in 1consomic), and a few where targets are unknown (*Taf5l, Nat8, Nat8b*). In certain cases, protein targets of the acetyltransferase have been identified: *Pafah1b1*, with targets platelet-activating factor and *Plekhm1*, and *Sat2*, with target eukaryotic initiation factor 5A, both lower in GK; and *Pafah1b3*, with target platelet-activating factor, *Phf20*, which targets histone; *Naa20*, which acts on proteins in cell cycle progression, and *Kat2a*, with target PGC-1 alpha, which are higher in GK. *Macrod1* is functionally related to *Sirt3*, because a product of *Sirt3* deacetylation reaction, O-acetyl-ADP-ribose, is a substrate of *Macrod1* [17]. *Macrod1* is not known to deacetylate proteins. Besides *Sirt3*, no other members of the sirtuin family are differentially expressed in liver (Table 3).

**Table 2:** Acetyltransferases in the rat liver transcriptome.

Symbol	Description	Illumina microarray				RNA-sequencing	
		GK/BN log2 fold-change	GK/BN FDR	1Cons/BN log2 fold-change	1Cons/BN FDR	GK/BN log2 fold-change	GK/BN FDR
*Pafah1b1*	platelet-activating factor acetylhydrolase 1b1	−1.69	**1.54E-09**	0.13	0.5895	0	1
*Sat2*	spermidine/spermine N1-acetyltransferase 2	−1.39	**2.32E-09**	−0.24	0.1951	−0.51	0.127
*Acat3*	acetyl-Coenzyme A acetyltransferase 3	2.00	**1.50E-07**	−0.02	0.9640	0.75	**0.0044**
*Phf20*	PHD finger protein 20	0.68	**1.73E-06**	−0.21	0.1457	−0.32	0.17198
*Nat8*	N-acetyltransferase 8	2.04	**3.42E-06**	0.62	0.1787	nf*	nf
*Nat1*	N-acetyltransferase 1	1.60	**8.05E-06**	0.47	0.2360	nf	nf
*Nat2*	N-acetyltransferase 2	−0.58	**0.0002**	0.07	0.7654	0.35	0.1461
*Pafah1b3*	platelet-activating factor acetylhydrolase 1b3	0.61	**0.0005**	0.14	0.5398	0.21	0.8658
*Taf5l*	TAF5-like RNA polymerase II	−0.59	**0.0005**	0.63	**0.0076**	−1.07	**8.87E-07**
*Gnpat*	glyceronephosphate O-acyltransferase	−0.29	**0.0017**	−0.12	0.3442	−0.29	0.2881
*Ncoa1*	nuclear receptor coactivator 1	−0.42	**0.0022**	−0.10	0.6087	0.03	0.9711
*Gnpnat1*	glucosamine-phosphate N-acetyltransferase 1	0.36	**0.0037**	0.06	0.7465	0.24	0.2677
*Naa20*	N(alpha)-acetyltransferase 20	0.27	**0.0039**	0.02	0.8875	nf	nf
*Kat2a*	K(lysine) acetyltransferase 2A	0.42	**0.0138**	0.09	0.7453	0.48	0.101
*Crat*	carnitine O-acetyltransferase	0.50	**0.0305**	−0.06	0.8800	1.06	**1.46E-11**
*Lpcat1*	lysophosphatidylcholine acyltransferase 1	0.06	0.6192	−0.43	**0.0146**	−0.34	0.533
*Nat8b*	N-acetyltransferase 8B	0.14	0.1751	−0.30	**0.0373**	−0.23	0.7107
*Lpcat3*	Lysophospholipid acyltransferase 5	nf	nf	nf	nf	0.51	**0.0047**
*Phf15*	protein Jade-2	nf	nf	nf	nf	0.77	**0.0043**

Shown are transcripts differentially expressed between BN and GK or 1consomic strains. False Discovery Rates (FDR)<0.05 are in bold.

**Table 3:** Liver deacetylases in the rat liver transcriptome.

Symbol	Description	Illumina microarray				RNA-sequencing	
		GK/BN log2 fold-change	GK/BN adjusted p-value	1Cons/BN log2 fold-change	1Cons/BN FDR	GK/BN log2 fold-change	GK/BN FDR
*Ndst1*	N-deacetylase/N-sulfotransferase 1	0.67	**8.26E-05**	0.056	0.8228	−0.04	0.9211
*Pigl*	phosphatidylinositol glycan anchor biosynthesis L	0.42	**0.0001**	−0.214	0.1077	nf*	nf
*Hdac5*	histone deacetylase 5	−0.75	**0.0007**	0.043	0.9096	0.13	0.6452
*Macrod1*	MACRO domain containing 1	−0.49	**0.0008**	0.286	0.1235	−0.56	**0.0018**
*Amdhd2*	amidohydrolase domain containing 2	−0.36	**0.002**	0.033	0.8696	−0.97	**0.0001**
*Hdac11*	histone deacetylase 11	−0.59	**0.0065**	−0.182	0.5643	−0.52	0.1061
*Aadac*	arylacetamide deacetylase	0.38	**0.0228**	0.434	0.0628	−0.49	0.0737
*Hdac3*	histone deacetylase 3	−0.22	**0.0345**	0.145	0.3341	nf	nf
*Atxn3*	ataxin 3	0.2	0.117	0.594	**0.0033**	0.42	0.0672
*Atxn1*	ataxin 1	nf	nf	nf	nf	0.93	**0.0001**
*Sirt1*	sirtuin 1	0.16	0.3331	−0.086	0.7452	0.14	0.7643
*Sirt2*	sirtuin 2	V	V	V	V	−0.19	0.4863
*Sirt3*	sirtuin 3	−0.74	**2.66E-06**	−0.928	**1.34E-05**	−0.66	**0.0008**
*Sirt4*	sirtuin 4	−0.15	0.1684	0.063	0.7183	−0.44	0.3751
*Sirt5*	sirtuin 5	0.12	0.3046	0.077	0.6704	0.1	0.8224
*Sirt6*	sirtuin 6	0.1	0.2433	0.129	0.2749	0.09	0.8905
*Sirt7*	sirtuin 7	−0.08	0.4437	−0.052	0.7549	0.31	0.3553

Shown are transcripts differentially expressed between BN and GK or 1consomic strains. False Discovery Rates (FDR)<0.05 are in bold (V: Variant in probe).

None of the differentially expressed acetyltransferases are in the chromosome 1 QTL linked to diabetes-related traits. Both acetyl-Coenzyme A acetyltransferase 3 and lysophosphatidylcholine acyltransferase 1 map to chromosome 1 but outside of the QTL region. Of the deacetylases tested for differential transcription, only *Sirt3* and *Macrod1* are within the QTL. As noted in Table S6, 40% of the acetylated proteins that we detected were also identified in a *Sirt3*-knockout model, and therefore are likely to be *Sirt3* targets [16]. We conclude that, although we observe some difference in other enzymes affecting acetylation, a significant fraction of the changes in lysine acetylation are likely to be due to down-regulation of *Sirt3*.

## Discussion

In this study, we have shown how transcript level and protein acetylation combine to control protein activity. This integrated, systems-level approach is crucial for understanding the regulation of complex phenotypes, including pathophysiological mechanisms involved in type 2 diabetes and cardiometabolic diseases. In our data, protein acetylation and gene transcription are independent of each other (figure 3) and hence have uncorrelated effects.

We identified a SNP in the promoter of *Sirt3* that is probably responsible for the lower expression of *Sirt3* in the liver of GK rats, and for downstream variation in protein acetylation in enzymes and pathways linked to diabetes and the metabolic syndrome. *Sirt3* transcription is strongly activated by PGC-1α in combination with ERRα in mouse muscle cells and hepatocytes [15]. It also mediates the influence of PGC-1α on mitochondrial biogenesis and the production of reactive oxygen species. Mouse knockouts of *Sirt3* cause mitochondrial protein hyperacetylation [12]. The effect of deacetylation by *Sirt3* on enzyme activity has been measured experimentally for 18 proteins [18], [19], and for 78% of these, the deacetylated form has higher activity. Enzymes whose activities increase upon deacetylation include mitochondrial acetyl-CoA synthetase (*AceCS2*), required for producing acetyl-CoA under ketogenic conditions such as fasting. *Sirt3* deacetylation also activates Complexes I (NDUFA9) and II (succinate dehydrogenase) of the electron transport chain, though SDH activity was reported to be affected only in MEFs and brown adipose tissue, not liver [10], [20]. *Sirt3^−/−^* knockout mice have reduced ATP production [21]. Other enzymes in the TCA cycle affected by deacetylation include isocitrate dehydrogenase (deacetylation causes higher activity) [22] and malate dehydrogenase (lower activity) [18]. *Sirt3* reduces reactive oxygen species in cells by activating mitochondria manganese superoxide dismutase 2 [23]. Mitochondrial 3-hydroxy-3-methylglutaryl CoA synthase 2 (*Hmgcs2*)[24], [25] glutamate dehydrogenase (*Gdh*) [22], forkhead box O3a (*Foxo3a*) [24], *Ku70* [25], long chain acyl CoA dehydrogenase (*Lcad*) [23], Serine/threonine protein kinase 11 (*Stk11*, or *Lkb1*) [26], and mitochondrial ribosomal protein L10 (*Mrpl10*) [27], are all activated when deacetylated by *Sirt3*. Of these proteins, we detect acetylation of succinate dehydrogenase and malate dehydrogenase in our data.

By integrating our GK and BN liver transcriptome and acetylome analyses, we can predict the effects on the TCA cycle (Figure 4). Malate dehydrogenase 1 (*Mdh1*), whose activity increases with acetylation, is only acetylated in BN liver, so BN *Mdh1* activity is predicted to be higher than in GK. Succinate dehydrogenase (*Sdh*; Complex II of oxidative phosphorylation) is only acetylated in GK liver. Since *Sdh* activity decreases when it is acetylated, we predict that GK *Sdh* activity is lower than in BN [27]. Isocitrate dehydrogenase 2 and fumarate hydratase transcripts are present at lower levels in GK than BN (though the subunits of a-ketoglutarate dehydrogenase complex are equivocal). Thus, the combined effect of lysine acetylation and transcript differences increases flux through the TCA cycle, and which should be higher in BN than GK liver.

Our results are consistent with a report showing that flux through Complex II with palmitoylcarnitine substrate is reduced in GK liver, but not in GK muscle [28]. We observe lower *Sirt3* transcript abundance with concomitant increased *Sdh* acetylation in liver, but no change of *Sirt3* transcription in muscle.

Studies of liver mitochondrial energetics in GK rats have demonstrated that the GK rat has a low endogenous ATP/ADP ratio [29], which could be the result of reduced flux through the TCA cycle. In the liver mitochondrial proteome, phosphoproteome and hydroxyproteome of GK rats, [30] suggest that protein expression is coordinated between the TCA cycle, fatty acid oxidation and oxidative phosphorylation during the progression from pre-diabetes to early Type 2 diabetes. Here we show that GK liver protein acetylation provides another layer post-translational control of enzyme activity. It is important to note that acetylation patterns in tissues other than liver are likely to be different [31].

We find that DNA variation in the *Sirt3* promoter may contribute to impaired glucose homeostasis in the GK model of type 2 diabetes. Diabetes in the GK rat is a complex, multifactorial disease, caused by susceptibility alleles isolated from an outbred Wistar stock upon selection of glucose intolerant animals. The polygenic threshold model for complex dichotomous conditions is therefore likely to apply [32]. Under this model, contributing factors accumulate until a threshold is reached, leading to descent into disease. Other rat strains may have reduced *Sirt3* deacetylase activity, but are below the threshold required to move into the disease state.

Our data also provide insights into the relationship between glucose intolerance and ageing. Wistar-related rat strains have shorter lifespans than BN rats [33]. Though there is some controversy about the role of sirtuins in ageing, they do mediate metabolic adaptation to stress and changes in nutrition [1] which affect ageing, and higher levels of *Sirt3* in BN rat may contribute to its longer lifespan. Furthermore, higher *Sirt3* in BN rat liver may contribute to its resistance to the metabolic syndrome. Knockdown of *Sirt3* in the BN rat and measurement of its effects on metabolism and longevity, could test this hypothesis.

## Materials and Methods

### Animals

GK, BN and congenic rat strains were provided from our breeding colony at Biomedical Services, University of Oxford. Rats were housed in groups of five per cage, with free access to water and standard laboratory chow pellets (B&K Universal Ltd., Grimston, Aldbrough, Hull) and were maintained on a 12-h light-dark cycle, at a room temperature of 21°C with a relative humidity of 55±10%. Tissues were collected from 7–8 month old animals that were fasted overnight and killed by a rising concentration of CO_2_. The tissue was flash-frozen and stored in a −80°C freezer until protein or RNA isolation was performed. Records of the breeding colony were kept using a FilemakerPro database [34]. Animal procedures were approved by the ethical review panel of the University of Oxford and UK Home Office project licence PPL 30/2918.

### RNA preparation

Liver and brown adipose tissue (BAT) were taken from six each of GK, BN.GK1consomic, and Brown Norway (BN control) male rats and flash-frozen in liquid nitrogen. Tissue samples were similarly taken from male BN.GK congenics derived for different sections of GK rat chromosome 1 (four each of strains BN.GK1b, 1h, 1o) (Figure 1B, right).

Total RNA was isolated from frozen tissues using either the RNeasy® kit (Qiagen, Crawley, UK), or Trizol reagent (Invitrogen Life Technologies, Paisley, UK). Briefly, for RNeasy kit, frozen tissue samples were homogenized in QIAzol Lysis Reagent using a Qiagen TissueLyser. Following phase separation after addition of chloroform, total RNA was purified using a spin technology according to the manufacturer's guidelines and eluted in RNase-free water. RNA was isolated from some tissues using Trizol reagent according to manufacturer's protocol. RNA concentrations were determined using a NanoDrop spectrophotometer and RNA quality, purity and integrity were assessed using an Agilent 2100 Bioanalyser (Agilent Technologies, Waldbronn, Germany).

### Illumina Bead Array hybridization and scanning

Gene transcription profiling for the BN, GK, and chromosome 1 congenic strains was performed using Sentrix® BeadChip RatRef-12 v1 Whole-Genome Gene Expression Arrays (Illumina Inc., San Diego, California, USA).

Double-stranded cDNA and purified biotin-labeled cRNA were synthesized from 300 ng high quality total RNA using the Illumina® TotalPrep RNA Amplification Kit (Ambion Inc., Austin, Texas, USA). cRNA concentrations were determined using a NanoDrop spectrophotometer whilst cRNA quality and integrity were assessed using an Agilent 2100 Bioanalyser (Agilent Technologies, Waldbronn, Germany). Hybridizations onto Sentrix® BeadChip RatRef-12 v1 Arrays were carried out using 750 ng of each biotinylated cRNA in a 58°C hybridization oven for 18 hours. Following washing and staining with Streptavidin-Cy3, the BeadChip Arrays were scanned on the Illumina® BeadArray Reader (Illumina Inc., San Diego, USA). Resulting data were then preliminarily analysed using the Illumina® BeadStudio Application software before undergoing comprehensive statistical analysis.

Microarray experiments were compliant with MIAME (Minimum Information About a Microarray Experiment) and both protocol details and raw data have been deposited in ArrayExpress (http://www.ebi.ac.uk/arrayexpress/) under the accession number E-MTAB-1048.

### Statistical analysis of Illumina microarray data and Gene Ontology annotation

Microarray data were imported and normalized using normexp [35]. Batch effects on expression values, as revealed by a principal component analysis, were dealt with by using the array identifier as additive covariant in the linear model. Differentially expressed genes were identified by comparing the 1consomic strain or GK with the BN strain. A linear model was fitted using the software LIMMA. Genes were tested for differential expression using a moderated t-statistic. Multiple testing correction of p-values was carried out using the false discovery rate [36].

### Quantitative real-time PCR

Total RNA was treated with Turbo DNA-free DNase kit, for removal of genomic DNA (Ambion Inc., Austin, Texas, USA). First-strand cDNA synthesis was performed using Superscript III First-strand Synthesis Supermix for qRT-PCR (Invitrogen, Paisley, UK). Assays were performed on a Rotor-Gene 3000 system (Corbett Research, Milton, UK) using the QuantiTect SYBR Green PCR kit (Qiagen Ltd., Crawley, UK), with PCR primers as listed in Table S1. Analysis was performed using the standard curve method (Rotor-Gene Software 5.0.47; Corbett Research, Milton, UK). Gene expression was normalized against the expression of either actin or acidic ribosomal phosphoprotein P0 (36B4). Experiments were performed in triplicate with samples prepared from 4–6 animals per group. Statistical significance was determined by the two-tailed independent sample t-test or univariate ANOVA, when testing more than two groups.

### Sequencing of the *Sirt3* gene and promoter cloning

Polymorphism information comparing GK and BN for the region containing Sirt3 and the flanking 5′ and 3′ sequence was obtained by comparing our genome sequence of the GK rat (Kaisaki, unpublished) with the BN reference sequence. BN-GK SNPs in the region of chr1:201019865-201045697 were submitted for analysis to the online tool at Ensembl, Variant effect predictor (http://www.ensembl.org/info/docs/variation/vep/index.html, [13]).

For cloning, the Sirt3 promoter region (RGSC3.4, chr1:201,043,757-201,044,402) was amplified with the primers Sirt3-promoter-F (5′-ACACAAATACCAGGCAGTCG-3′) and Sirt3-promoter-R (5′-ACCGTTGACAGCTTATCTGC-3′) from genomic DNA of either GK or BN rats, using proofreading enzyme Phusion High Fidelity PCR mix (New England Biolabs, Hitchin, UK), and ligated first into PCRII-TOPO vector (Invitrogen, Paisley, UK) with Roche Rapid Ligation kit, and then subcloned into pGL3-basic Luciferase Reporter vector (Promega, Southampton, UK). Clones were sequenced to confirm there were no PCR errors.

### Cell transfection and luciferase assay

HEP3B hepatoma and HEK293T cells were plated in 24-well plates at 2.5×10^4^ cells/well, and transfected the next day using Fugene 6, following manufacturer protocols (Roche Molecular Biochemicals, Burgess Hill, UK). Two different concentrations of pGL3-promoter plasmid were transfected, and pCMV-LacZ was co-transfected in all cells (pCMV-LacZ was kindly provided by J. Braganca and S. Bhattacharya). Luciferase activity was measured using Promega Luciferase Assay System (Promega, Southampton, UK), and read on a Labsystems Luminoskan Ascent microplate luminometer (Thermo Fisher Scientific, Loughborough, UK). Activity of β-galactosidase was assayed by measuring cleavage of the substrate *o*-nitrophenyl-β-D-galactoside (ONPG) [37]. Two to three independent transfection experiments were run, each time in triplicate wells, and results are corrected for transfection efficiency by expressing data as the ratio of luciferase to β-galactosidase activity.

### Analysis of acetylated proteome

#### Protein extraction and trypsin digest

Detection of protein acetylation was previously shown to be more optimal when the enrichment step as performed at the peptide level [31], [38]. Liver tissue was homogenised in Lysis buffer, consisting of 50 mM Tris-HCl, pH 7.4, 0.5% NP-40 substitute, 150 mM NaCl, 20 mM MgCl_2_, 10 µM trichostatin A, 10 mM nicotinamide, 50 mM butyric acid, and protease inhibitor cocktail (Pierce, Cramlington, UK), using a Tissuelyser at 20 mHz for 3 min (Qiagen Ltd., Crawley, UK). Samples were rotated in a cold room for 30 min, sonicated, and centrifuged 13000 rpm for 20 min at 4°C to remove solids. Protein concentration was measured (Pierce BCA protein assay kit, Perbio Science UK, Ltd, Cramlington, UK), and 30 mg of protein from each sample was methanol-chloroform extracted [39]. The pellet was resuspended in 6 M urea in 0.1 M Tris, pH 7.8 by vortexing and sonicating. The proteins were reduced in 10 mM DTT, alkylated with 40 mM iodoacetamide, and reduced again in 40 mM DTT. Urea concentration was reduced to 0.7 M by diluting with water, then trypsin was added in a 1∶50 ratio (i.e. 60 µg trypsin for 30 mg protein), and incubated at 37°C overnight. Samples were desalted using Sep-pak C18 cartridges (Waters Ltd, Elstree, UK), then evaporated to dryness in a speed-vac.

#### Immunoprecipitation of acetylated peptides

Pellets were resuspended in RIPA buffer (50 mM Tris, pH 7.4, 150 mM NaCl, 1% NP-40 substitute (Sigma-Aldrich, Gillingham, UK), 0.25% Na-deoxycholate, 1 mM EDTA), then diluted in NET buffer (50 mM Tris, pH 7.4, 5 mM EDTA, 150 mM NaCl). The tryptic peptides were cleared of non-specifically binding peptides by incubating with Protein A-dynabeads (Invitrogen, Paisley, UK). Immunoprecipitation was performed using a mixture of mouse monoclonal and rabbit polyclonal pan-acetyllysine antibodies (NEB Cell Signalling Technology, Hitchin, UK) conjugated to Protein A-dynabeads in NET buffer. Beads were washed three times with NET buffer, transferred to a fresh tube, and eluted twice with ice-cold 100 µl elution buffer (100 mM glycine, pH 2.5). Eluates were desalted using Supel-Tips C18 micropipette tips (“zip-tips”, Sigma-Aldrich, Gillingham, UK), and evaporated to dryness in a speed-vac.

#### LC-MS/MS Mass-spectrometric analysis

The analysis of digested immunoprecipitated material was performed by LC-MS/MS using an orbitrap Velos (Thermo) coupled to a nano-UPLC system (NanoAcquity, Waters) using a reversed phase 75 µm×250 mm C18 column as described [39]. MS/MS spectra were searched against the NCBInr Rodentia database (v2012.07.08, 18970916 sequences) using the Mascot search engine v2.3.01, allowing two missed cleavage and 20 ppm/0.5 Da mass deviations in MS/MSMS, respectively. Carbamidomethylation of cysteine was a fixed modification. Oxidation of methionine, and acetylation of lysine were used as variable modifications. Acetylated peptides were identified on a Mowse score probability based scoring algorithm with a >95% confidence of identification. Acetylated lysine residues were detected as +42.01 Da mass tags and MS/MS spectra required at least four consecutive b or y ions. Representative MS/MS spectra of acetyl-lysine containing peptides are shown in Figure S2, and the peptide scores for each acetylated peptide observed in either the GK or BN samples were compared for an indication of a change in relative abundance (Table S6).

### RNA-sequencing

RNA-sequencing was performed according to standard operating procedures by the High-Throughput Genomics Group at the Wellcome Trust Centre for Human Genetics. Briefly, mRNA was selected from the total RNA, then fragmented and converted to cDNA. The cDNA was end-repaired, A-tailed and adapter-ligated before amplification and size selection. The prepared libraries were multiplexed and quality controlled before 51-nt paired end sequencing on an Illumina HiSeq2000 next generation sequencing machine.

Sequencing reads were mapped to the rat reference genome (RGSC3.4, Ensembl release 69) using tophat version 2.0.6 [40]. Bam files were filtered using samtools version 0.1.17 [41], removing alignments with MAPQ <15. Filtered Bam files were sorted and duplicates were removed using samtools. Differential expression was detected with edgeR [42]. Significant enrichment of KEGG pathways was analysed with goseq [43].

## Supporting Information

Figure S1 **Luciferase reporter assays for transfection of kidney cell line.**
(PDF)

Figure S2 **Mass spectrometry based detection of protein lysine acetylation.**
(PDF)

Table S1 **Variant Effect Predictor for SNPs and indels in the region of** ***Sirt3*** **.**
(XLS)

Table S2 **RNA-sequencing differentially expressed genes in brown adipose tissue of GK and BN rats.**
(XLS)

Table S3 **RNA-sequencing differentially expressed genes in liver of GK and BN rats.**
(XLS)

Table S4 **Significant enrichment of KEGG pathways in brown adipose tissue.**
(XLSX)

Table S5 **Significant enrichment of KEGG pathways in liver.**
(XLSX)

Table S6 **Acetylated peptides in GK and BN rat liver, and their transcriptional expression.**
(XLS)
