# Supplementary material for: Genetic Control of Differential Acetylation in Diabetic Rats
Source: PLoS One. 2014 Apr 17;9(4):e94555. doi: 10.1371/journal.pone.0094555 (PMC3990556; doi:10.1371/journal.pone.0094555)
Supplement: Figure S1 — Luciferase reporter assays for transfection of kidney cell line. (PDF) [file pone.0094555.s001.pdf]

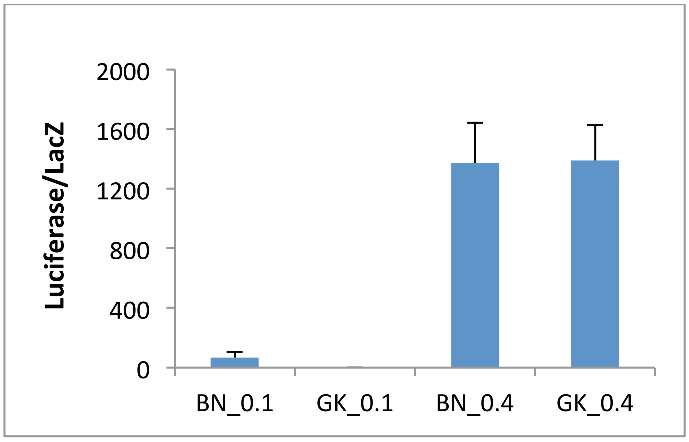

Supplementary Figure 1. Luciferase reporter assay results for transfection of kidney cell line. Clones of BN or GK allele of Sirt3 promoter in pGL3-basic vector were transfected into HEK293T cells at two levels, 0.1 and 0.4ug/well (not significantly different). Results are corrected for transfection efficiency by co-transfection with beta-galactosidase vector, and represent two-three independent transfections, which were run in triplicate wells.
