# Supplementary material for: Genetic Control of Differential Acetylation in Diabetic Rats
Source: PLoS One. 2014 Apr 17;9(4):e94555. doi: 10.1371/journal.pone.0094555 (PMC3990556; doi:10.1371/journal.pone.0094555)
Supplement: Figure S2 — Mass spectrometry based detection of protein lysine acetylation. (PDF) [file pone.0094555.s002.pdf]

A

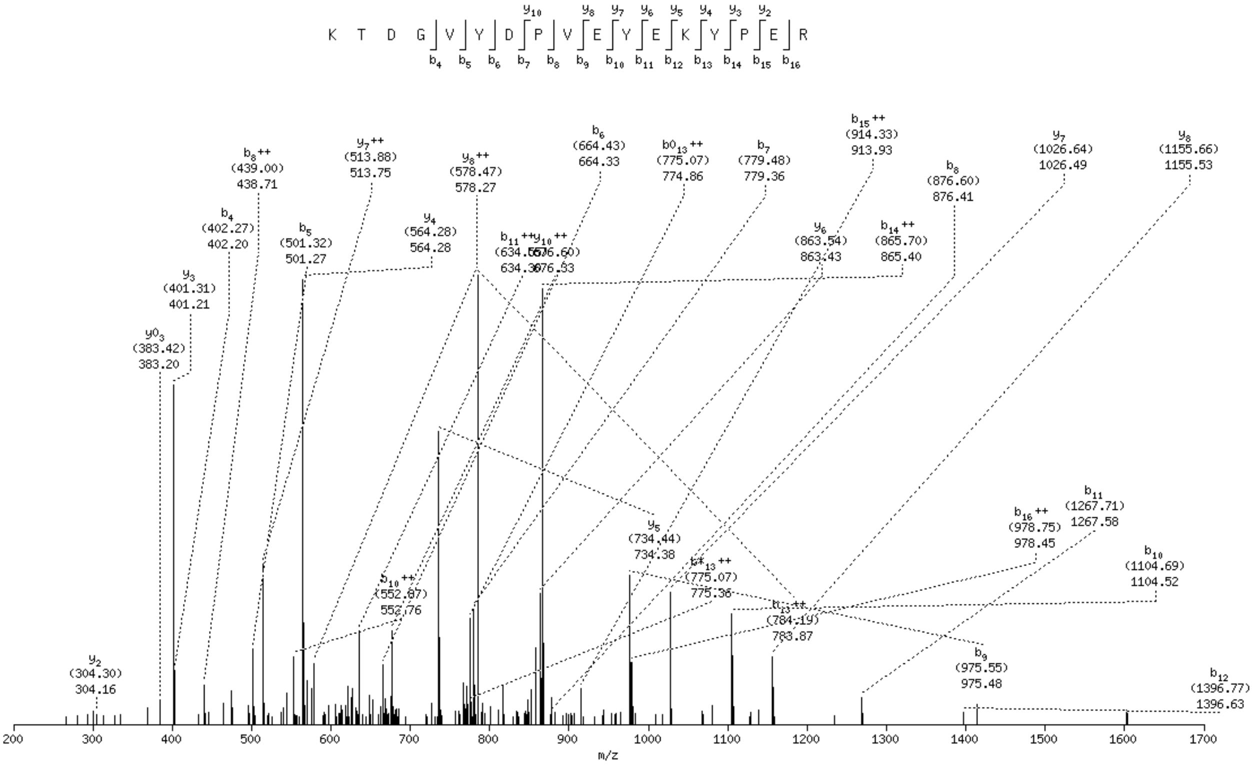
K T D G V Y D P V E Y E K Y P E R
m/z
200
300
400
500
600
700
800
900
1000
1100
1200
1300
1400
1500
1600
1700

B

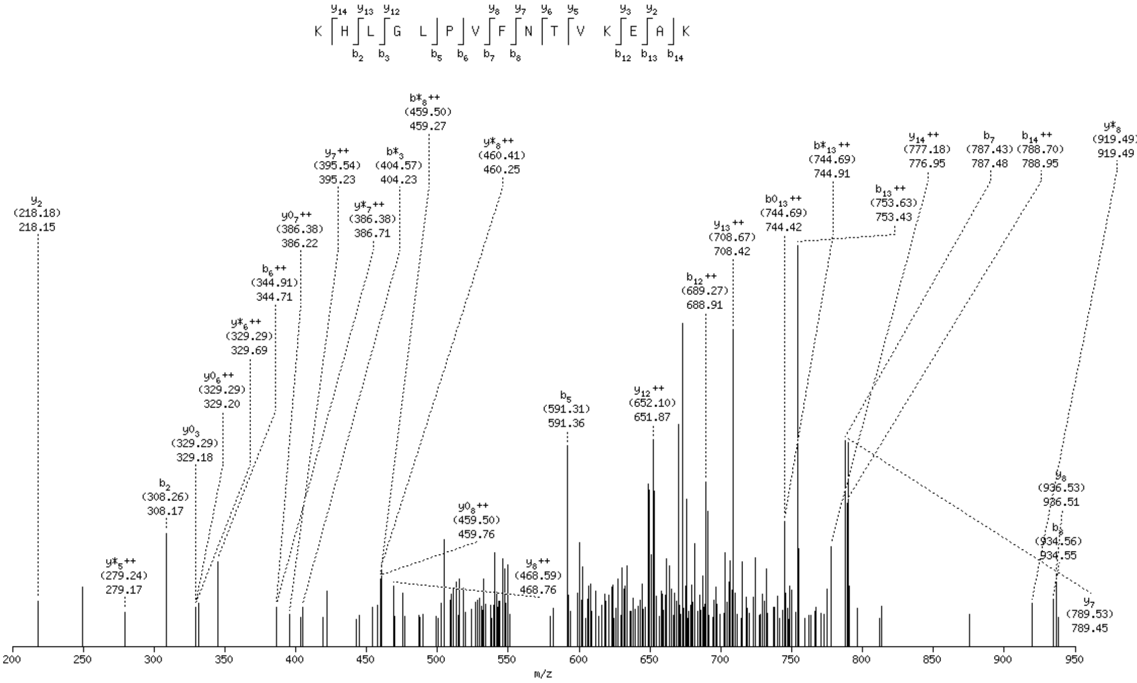
K H L G L P V F N T V K E A K
m/z
200
250
300
350
400
450
500
550
600
650
700
750
800
850
900
950
